# Recurrent Meningitis in the Context of an Encephalocele

**DOI:** 10.7759/cureus.29594

**Published:** 2022-09-26

**Authors:** Kimberly Go, Jiazeng Ge, Mohammed Abdelattif, May Zaw

**Affiliations:** 1 Internal Medicine, American University of the Caribbean School of Medicine, Cupecoy, SXM; 2 Internal Medicine, BronxCare Health System, New York City, USA; 3 Internal Medicine, Icahn School of Medicine at Mt. Sinai, New York City, USA

**Keywords:** negative csf culture, spontaneous rhinorrhea, streptococcus pneumoniae, encephalocele, recurrent meningitis

## Abstract

Recurrent meningitis in adults with an encephalocele is an uncommon occurrence. We present a case of a 57-year-old female with recurrent meningitis upon a new discovery of a sphenoidal encephalocele. In this case, the patient did not exhibit recurrent meningitis until 10 years after a fall injury not associated with direct head trauma. However, her fall did result in a temporary loss of consciousness. She began to have spontaneous intermittent cerebrospinal fluid (CSF) rhinorrhea and headaches throughout the following 10 years without any diagnosis of meningitis. We discuss the causes of subsequent recurrent meningitis associated with CSF leakage and encephaloceles.

## Introduction

The definition of recurrent meningitis remains widely debated. The majority have defined it as two or more episodes of meningitis caused by either different organisms or the same organism upon having more than three weeks post-completion of therapy [1-2]. Recurrent meningitis usually occurs due to an anatomical defect or immunological defense deficiency. The anatomical defects can either be congenital or acquired through trauma [3]. One of these rare congenital anatomical defects is an encephalocele, a protrusion of the brain tissue through an opening in the skull [2]. Recurrent meningitis also occurs in patients with a history of head trauma, often leading to cerebrospinal fluid (CSF) rhinorrhea or leakage. CSF leakage is considered an important risk factor for bacterial meningitis. In a prior study, 38% of patients with recurrent meningitis had reported CSF leakage. Patients with CSF-leakage-associated meningitis often have a high recurrence rate despite vaccination and surgical repairs [4]. CSF rhinorrhea has also appeared within hours to months from the time of head injury. Subsequently, recurrent bacterial meningitis tends to occur within the first three months [5]. The most common predisposing factors in recurrent meningitis are remote head injury and CSF leakage [1].

The most common organisms that cause meningitis are* Streptococcus pneumoniae*, *Haemophilus influenzae*, and *Neisseria meningitidis*. However, CSF-leakage-associated meningitis is most commonly associated with *Streptococcus pneumoniae* and *Haemophilus influenzae* [2,6]. Group B Streptococcus (GBS), otherwise known as *Streptococcus agalactiae*, is usually the cause of meningitis in neonates and children less than three years old. While GBS meningitis accounts for only 1.3% of meningitis cases in adults, it is still possible. Adults with meningitis due to *Streptococcus agalactiae* tend to be elderly or have a chronic disease such as diabetes mellitus [7]. *Streptococcus pneumoniae *remains the most common cause, especially in cases of recurrent meningitis [8].

According to Ter Horst et al., there is an approximate 20% mortality rate from community-acquired meningitis in high-income countries. Nevertheless, early intervention, such as empiric antibiotic treatment, plays a valuable role in favorable outcomes. Patients with recurrent bacterial meningitis often have better results than those with nonrecurrent meningitis. This difference may be due to greater awareness of the signs and symptoms seen in meningitis [6]. Overall, recurrent meningitis remains a critical issue addressed in the clinical setting.

## Case presentation

We report the case of a 57-year-old, Hispanic female patient who presented to the emergency department with a persistent, worsening right frontal headache radiating to the jaw along with nausea and vomiting for three days with little relief from Tylenol and naproxen. The patient presented with altered mentation, fever, and tachycardia during this admission. Vital signs included a temperature of 103°F, pulse of 116 beats per minute, blood pressure of 130/70 mmHg, and respiration rate of 16 breaths per minute. On physical examination, the patient appeared drowsy. She did not follow commands but responded to verbal stimuli and presented with spontaneous limb movement. Her head appeared normocephalic and atraumatic. The neck was supple. The pupils were round, regular, and reactive to light. She denied having any visual changes. There was no edema, cyanosis or clubbing noted.

Her past medical history included diabetes mellitus, hypertension, and asthma. She had one episode of bacterial meningitis with CSF leakage and seizures that occurred one year before this hospital visit. Group B Streptococcus was determined to be the cause of the first case of meningitis. She had reported recurrent clear nasal secretions and headaches over 10 years since having a traumatic fall in which she landed on her coccyx area. At that time, she did not hit her head but temporarily lost consciousness afterward. The amount of rhinorrhea changed based on the season, with increased amounts during the winter. She denied having any prior head surgery and any recent travel. She also denied any history of tobacco, alcohol, or illicit drug use.

**Figure 1:**
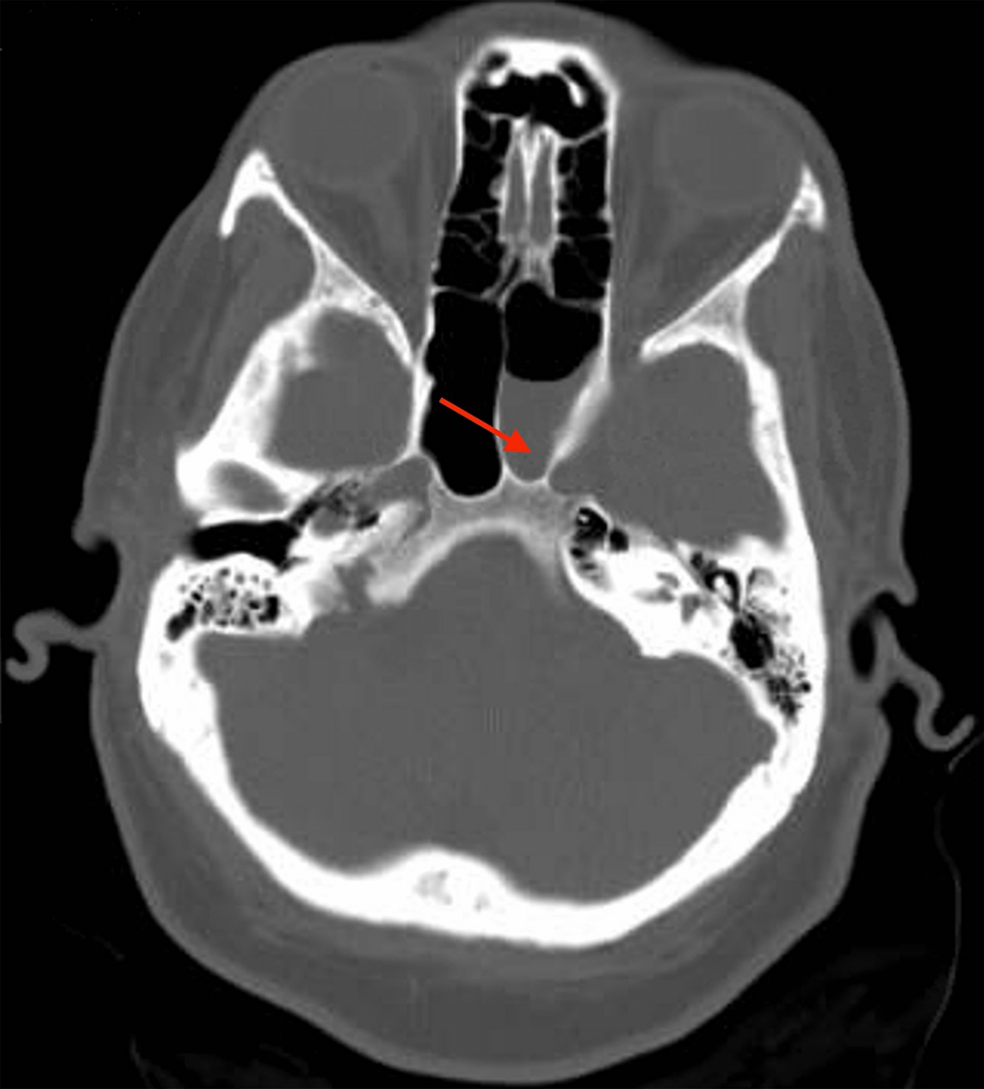
Axial computed tomography (CT) of the head and sinus Axial CT of the head and sinus depicts increased opacification (red arrow) in the left sphenoidal sinus

**Figure 2:**
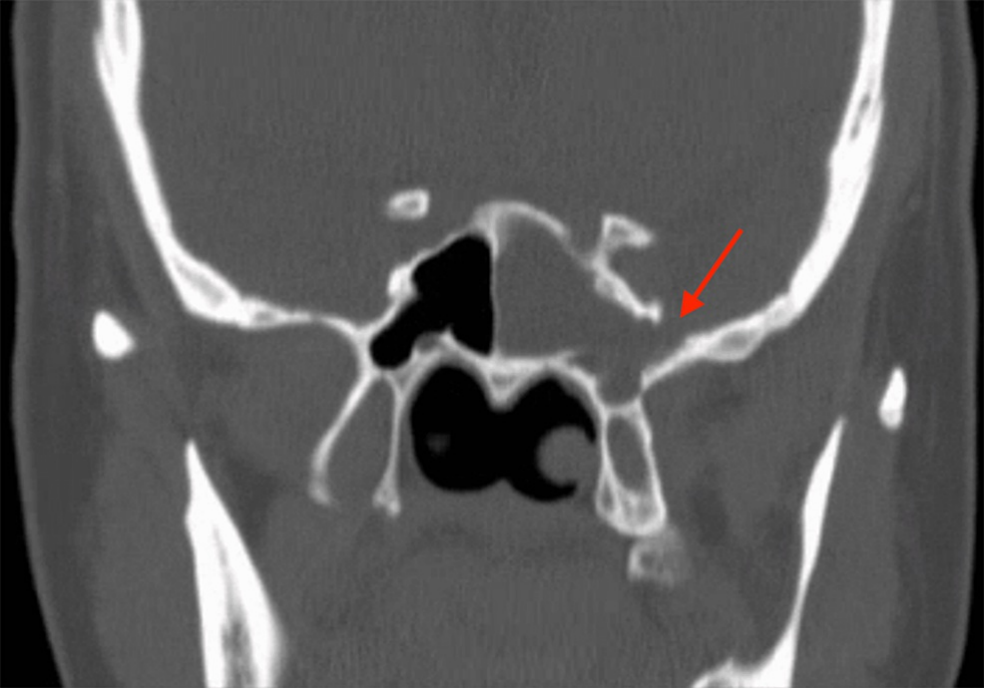
Coronal computed tomography (CT) of the head and sinus As depicted by the red arrow, the coronal CT of the head and sinus showed soft tissue in the lateral aspect of the left sphenoid sinus that appears to communicate with the inferior aspect of the left temporal lobe through a bone defect.

Because of previous meningitis and allergy to ceftriaxone, the patient started empiric treatment for meningitis. The treatment consisted of intravenous vancomycin, sulfamethoxazole with trimethoprim, levofloxacin, and acyclovir. She also received doses of 10 mg intravenous dexamethasone completed over four days. The white blood cell (WBC) count was 10.6 k/μl with a high neutrophil percentage of 85.0%. The C-reactive protein level was elevated at 5.78 mg/dL. The initial computerized tomography (CT) of the head showed no acute changes. The patient then underwent a lumbar puncture. CSF analysis revealed milky, cloudy fluid with a WBC count of 4,370 (neutrophil 98% and lymphocytes 2%), a protein level of 305 mg/dL, and a glucose level of 35 mg/dL. CSF culture showed few white blood cells but no organism growth. Cryptococcal antigen and Herpes Simplex Virus (HSV) tests were negative. Hence, acyclovir was discontinued. The blood culture was positive for *Streptococcus pneumoniae*. Immunoglobulin class and complement levels were within the normal range. A beta-2 transferrin test from the clear nasal discharge was also positive, which confirmed the presence of CSF leakage. The CT of the head and sinus (Figure 1) was completed and showed increased opacification in the left sphenoid sinus. As seen in Figure 2, the soft tissue in the lateral aspect of the left sphenoid sinus appears to communicate with the inferior aspect of the left temporal lobe through a bone defect. This image result indicated a congenital abnormality with an encephalocele formation.

Our patient’s mentation improved after being treated with intravenous antibiotics. Because of CSF leakage, the patient underwent sinus surgery and a pterional craniotomy after treatment of the infection. Postoperatively, she initially reported difficulties keeping her right eye open in addition to headaches, photosensitivity, and vision changes. However, those symptoms have improved over time.

## Discussion

Encephalocele, a herniation of the brain tissue, is a rare congenital anatomical defect that may result in bacterial invasion of the meninges [2]. The cause of encephaloceles is multifactorial and involves multiple sites of anterior neural tube closure [9]. The incidence of encephaloceles is approximately one in 10,000 live births worldwide [10]. The survival probability of children born with encephalocele to the age of 20 was 67.3%, and worldwide hospital-based studies suggested that the survival range was 50-95% in 2003 [9]. The most common location of an encephalocele is in the occipital region. Basal encephaloceles are extremely rare and only account for about 1.5% of all encephaloceles. There are four types of basal encephaloceles: transsphenoidal, spheno-orbital, sphenoethmoidal, and transethmoidal. A transsphenoidal encephalocele may be further classified into the true transsphenoidal and intrasphenoidal types, depending on the extent of the encephalocele through the sphenoid sinus. Diagnosing an intrasphenoidal encephalocele can be difficult because of its rarity and nonspecific signs and symptoms. The failure to recognize the lesion may result in meningitis, as seen in our patient, or even death [11].

Brain tissues exposed to the sinuses or external environments are at high risk of infection. Adults who present with spontaneous CSF rhinorrhea should be suspected of an intracranial encephalocele as it may be a late presentation [12]. Cases of spontaneous CSF rhinorrhea in literature, particularly in those who were female or middle-aged, have frequently been associated with encephalocele in the rhino-ethmoid or sphenoid sinus region [13]. Two sensitive and specific confirmatory tests for CSF rhinorrhea are the presence of either beta-2 transferrin or beta-trace protein in the nasal discharge [8]. Our patient has a positive result for beta-2 transferrin, which further confirms her CSF rhinorrhea.

As previously mentioned, CSF leakage is associated with meningitis. Our patient had a positive blood culture for *Streptococcus pneumoniae* but a negative CSF culture. We cannot confirm *Streptococcus pneumoniae* as the infectious cause of her recurrent meningitis because she received antibiotics before completing the lumbar puncture; thus, a negative CSF culture could result. The CSF culture sensitivity decreases when antimicrobials are administered in general [14]. There was a report of 11-41% of patients clinically suspected of bacterial meningitis with negative CSF cultures [6]. Hence, a negative CSF culture may be more common in recurrent meningitis. In addition, her CSF cell count and differential indicated an elevated WBC count of 4,370 with neutrophilic predominance, an elevated protein level of 305 mg/dL, and a decreased glucose level of 35 mg/dL. These results and a cloudy CSF appearance are highly suggestive of bacterial meningitis. In a study, CSF protein concentration was determined to have the highest sensitivity, specificity, and accuracy for bacterial meningitis. A high CSF protein content of over 50 mg/dL, cytochemical CSF analysis, and a clinical presentation of meningitis can be used for diagnosing bacterial meningitis [14]. Given the prior history of meningitis due to *Streptococcus agalactiae*, this patient also meets the criteria of recurrent meningitis.

Recurrent meningococcal meningitis is associated with immune deficient states involving the complement system and agammaglobulinemia [15]. However, our patient’s immunoglobulin class and complement levels were within the reference range, ruling out possible immune deficiency states causing her recurrent meningitis. Recurrent pyogenic meningitis likely implies an anatomical communication between the CSF space and the nonsterile environment. Communication can be congenital or acquired. Congenital defects typically involve the skull base or middle ear, although persistent dermal sinuses can be found anywhere along the vertebral column [15]. Acquired defects are most commonly seen in head trauma or surgeries. Closed head trauma can be a reason for recurrent meningitis years later. There is a case report about a 33-year-old, otherwise healthy male presenting with community-acquired meningitis who was later found to have a remote history of closed head trauma and CSF rhinorrhea for years. Imaging showed that he had a skull base defect [16]. Even though a traumatic fall injury could be a potential cause of closed head injury, she denied any injury to the head, thus ruling out closed head injury as a cause. She also did not exhibit recurrent meningitis until 10 years after her traumatic fall injury, making acquired defects an unlikely cause. Her presentation of a congenital encephalocele on the CT head and sinus is the most likely cause of her recurrent meningitis.

The prognosis of an encephalocele depends on the location, the size of the defect, the amount of brain tissues inside the sac, and the presence of hydrocephalus. The prognosis is better for patients with frontoethmoidal encephaloceles than those with parietal or occipital encephaloceles. Hydrocephalus, brain tissue in the sac, and the presence of other intracranial abnormalities are poor prognostic factors in patients with an encephalocele [17]. Brain abscess, a possible complication of congenital encephalocele, may be a concerning cause for such brain herniation and hydrocephalus. In addition, post-neurosurgical procedures and trauma are considered major predisposing factors to brain abscess development. According to research, a brain abscess is associated with a high mortality risk even after patients have resolved from acute infection. There is a 21% mortality rate after a year, a 16% mortality rate in two to five years, and an even greater mortality rate of 27% after six to 30 years [10,18]. While our patient would have a postoperative risk for brain abscess, her initial prognosis regarding the congenital encephalocele was favorable. The location of her encephalocele, the lack of hydrocephalus upon presentation, and the onset of symptoms occurring between the ages of 50 and 60 all suggest a good prognosis.

## Conclusions

In conclusion, our patient’s recurrent meningitis was most likely the result of her congenital encephalocele. Her lab findings and history suggest that this was a case of recurrent meningitis rather than an individual case of acute meningitis. Thus, a chronic condition was the most likely cause. All the other possible causes of recurrent meningitis, such as immune deficiency and head trauma, were ruled out by past medical history and lab findings. Her prompt recovery after the pterional craniotomy additionally supports our conclusion. While this case report highlights the role of encephaloceles in recurrent meningitis, its limitations include the finite information provided by the patient to the clinicians and its focus on an individual experience. Finally, the study of recurrent meningitis associated with encephaloceles would benefit from a meta-analysis in the future.
